# MINIMALLY INVASIVE OSTEOSYNTHESIS FOR CLAVICULAR FRACTURE WITH LOCKED PLATE

**DOI:** 10.1590/1413-785220233102e263742

**Published:** 2023-05-01

**Authors:** FELIPE MACHADO DO AMARAL, EDUARDO ANGELI MALAVOLTA, FERNANDO BRANDÃO DE ANDRADE E SILVA, LEANDRO SOSSAI ALTOÉ, CASSIO VELLOSO NUNES, JOSÉ RICARDO PÉCORA

**Affiliations:** 1 Universidade de Sao Paulo, Hospital Universitário, Sao Paulo, SP, Brazil.; 2 Universidade de Sao Paulo, Hospital das Clínicas, Faculdade de Medicina, Sao Paulo, SP, Brazil.; 3 Hospital do Coração, Sao Paulo, SP, Brazil.; 4 Hospital Geral de Pedreira, Sao Paulo, SP, Brazil.; 5 Universidade de Sao Paulo, Hospital Universitário, Departamento de Ortopedia, Sao Paulo, SP, Brazil.

**Keywords:** Clavicle. Fractures, Bone. Osteosynthesis, Clavícula, Fraturas Ósseas, Osteossíntese

## Abstract

**Objective:**

To evaluate the clinical and radiographic results of the surgical treatment of fractures of the middle third of the clavicle, using the technique of minimally invasive plate osteosynthesis (MIPO) with locking.

**Methods:**

Prospective case series, evaluating displaced fractures of the middle third of the clavicle submitted to MIPO with locking, with procedures performed by a single surgeon. Patients were evaluated at 12 months using the University of Los Angeles (UCLA) scale and anteroposterior radiographs of the clavicles with 45° cranial and caudal inclination, as well as reporting complications.

**Results:**

In total, 15 patients were evaluated. The median of surgical time was 50 minutes (IQR 35). The UCLA scale had a median of 35 (IQR 2) at 12 months. All patients presented fracture healing. Minor complications occurred in three cases (20%), with two (13.3%) evolving with plate prominence and one (6.7%) with local paresthesia, while major complications occurred in only one case (6.7%), with suture dehiscence requiring surgical re-approach.

**Conclusion:**

MIPO with locking is a viable option for the treatment of displaced fractures of the middle third of the clavicle, with excellent results according to the UCLA scale, fracture healing in all cases, and a low rate of complications. ***Level of Evidence IV, Case Series.***

## RESUMO

**Objetivo:**

Avaliar os resultados clínicos e radiográficos do tratamento cirúrgico de fraturas do terço médio da clavícula pela técnica de osteossíntese minimamente invasiva com placa (OMIP) bloqueada.

**Métodos:**

Série de casos prospectiva que avaliou fraturas desviadas do terço médio da clavícula submetidas à OMIP bloqueada, com procedimentos realizados por um único cirurgião. Os pacientes foram avaliados aos 12 meses por meio da escala da Universidade da Califórnia em Los Angeles (UCLA) e por radiografias das clavículas em anteroposterior (AP) com inclinação cranial e caudal de 45°, além de relatos de complicações.

**Resultados:**

Foram avaliados 15 pacientes. A mediana do tempo cirúrgico foi de 50 minutos (IIQ 35). A escala da UCLA aos 12 meses teve mediana de 35 (IIQ 2). Todos os pacientes apresentaram consolidação da fratura. Complicações menores ocorreram em três casos (20%): dois (13,3%) com proeminência da placa e um (6,7%) com parestesia local. Maiores complicações ocorreram em apenas um caso (6,7%), com deiscência de sutura, necessitando de reabordagem cirúrgica.

**Conclusão:**

A OMIP bloqueada se mostrou uma opção viável ao tratamento das fraturas desviadas do terço médio da clavícula, com resultados excelentes de acordo com a escala UCLA, consolidação em todos os casos e baixo índice de complicações. **Nível de Evidência IV, Série de Casos.**

## INTRODUÇÃO

Clavicle fracture represents 44% of fractures of the scapular waist and 2.6% of fractures of the human body. They are more frequent in young and active individuals. The main mechanism of trauma of clavicle fracture results from a fall on the shoulder, in a smaller frequency by indirect trauma on the outstretched arm or direct trauma to the clavicle.^1^ ^)- (^ ^3^

Fractures of the middle third of the clavicle are the most common, representing 80% of clavicle fractures. ^(^ ^1^ ^), (^ ^3^ Although the most show good results with the conservative management, pseudarthrosis rates are higher than those treated surgically. ^(^ ^4^ ^), (^ ^5^ Displaced fractures and fractures without contact between fragments present faster fracture healing, better functional results, and lower rates of complications when treated surgically. ^(^ ^3^ ^), (^ ^6^

Osteosynthesis with open plate is considered the gold standard in the surgical treatment of fractures of the middle third of the clavicle. ^(^ ^4^ ^), (^ ^5^ Some authors advocate the minimally invasive plate osteosynthesis (MIPO) with locking as a way to reduce complications and surgical aggression to the fracture focus, as well as to improve the aesthetic aspect. ^(^ ^7^ However, few studies have been published on this subject. ^(^ ^7^ ^), (^ ^8^

### Objectives

This study aimed to describe the functional and radiographic results, as well as the complications of the MIPO with locking technique for fractures of the middle third of the clavicle.

## METHODS

**Figure 1:**
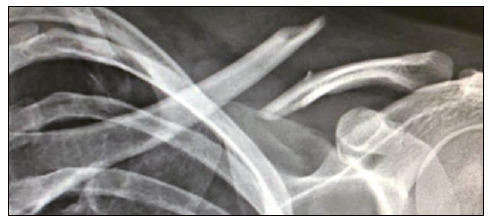
Complete transverse and displaced fracture of the middle third of the clavicle.

A series of prospective cases of patients with fracture of the middle third of the clavicle were performed and subjected to MIPO with locking. All procedures were performed in the same institution by a single surgeon, member of the Brazilian Society of Shoulder and Elbow Surgery (SBCOC) and the Brazilian Society of Orthopedics and Traumatogia (SBTO), and with seven years of experience, between January 2017 and December 2018. The study was approved by The Ethics Committee (229891 19.3.0000.8054), and the patients signed an informed consent form. Inclusion criteria were: skeletally mature patients, between 18 and 70 years old, and with displaced fractures of the middle third of the clavicle. As a deviation, the presence of fracture without bone contact, with ≥ 2 cm, intermediate displaced fragment (Z-fragment), or tent skin were considered (Figure 1). Exclusion criteria were: fractures older than two weeks between trauma and the surgical procedure, fractures that included the proximal and/or distal third of the clavicle, exposed or pathological fractures, any previous morbidity of the affected upper extremity that could compromise limb function, bilateral clavicle fractures, or previous history of ipsilateral fracture, and presence of neurovascular involvement in the initial trauma.

The time of surgery was evaluated, and the moment of incision was standardized until the last stich on the skin was closed.

Clinical evaluation was performed by the scale of the University of California (UCLA), Los Angeles, and applied by the surgeon at 12 months of follow-up.^9^ ^), (^ ^10^

All patients initiated pendulum movements, encouraging active and passive movement in the immediate postoperative period. Patients started physical therapy after six weeks, twice a week. In total, 20 sessions were performed, the last ten with resistance active exercises.

Radiographic evaluation was performed with anteroposterior (AP) radiographs, cephalic inclination of 45°, and caudal of 45° of the clavicle at two and six weeks, and at three, six, and 12 months after the surgery. Fractures with at least three integrated corticals were considered consolidated. Absence of fracture healing after three months was considered a delay in fracture healing, and at six months, pseudarthrosis.

Complications were divided into greater or minor, according to the impact on patient function or increased treatment time. Thus, the following events were evaluated:

Minor complications: hypertrophic scar, peri-incisional paraesthesia, skin irritation, or prominence of osteosynthesis material.

Major complications: osteosynthesis failure, refracture, superficial infection or suture dehiscence requiring reoperation, deep infection, and consolidation failure.

### Surgical technique

**Figure 2:**
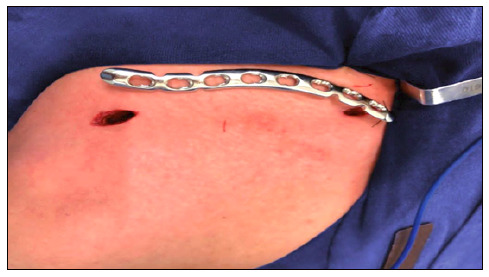
Measurement of plate size and percutaneous pathway access.

The patients were subjected to general anesthesia associated with interscalene block of the brachial plexus and positioned in dorsal decubitus in the beach chair position. Initially, the plate size is chosen, leaving a minimum work area of four holes, with the help of radioscopy. After demarcation of the length of the plate on the skin, two 1.5-cm accesses are made on the distal and proximal parts of the plate equidistant to the fracture trait (Figure 2).

Then, subcutaneous tissue detachment was performed in the upper aspect of the clavicle, incision of the fascia, with maximum periostal preservation possible until the communication of the two accesses, medial and lateral. The plate was placed by the medial access toward the lateral access.

**Figure 3:**
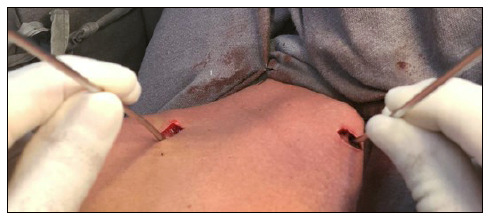
Intraoperative image of the joystick technique to indirectly reduce the fracture.

For fracture reduction, two 2.0-mm Kirschner wires are inserted into the anterior surface of the clavicle by each of the access, medial and lateral, and the deviation was reduced by using the joystick technique (Figure 3). A 2.0-mm Kirschner wire is inserted into the most dislocated holes of the plate at the fracture focus, medial and lateral, for temporary fixation of the plate.

**Figure 4:**
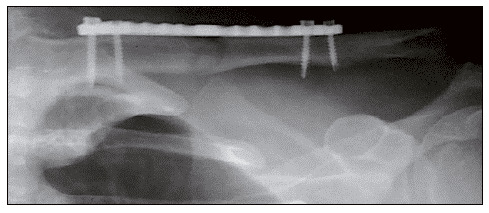
Radiography in postoperative anteroposterior incidence showing fracture reduction and fixation by plate and screws.

A cortical screw is inserted centrically into each bone fragment, close to the fracture focus. A 3.5-mm blocking screw is inserted into each bone fragment in the previous location of the Kirschner wire (Figure 4).

The closure of the muscular fascia is performed with Vycril 1 wire, with continuous anchored point, the subcutaneous tissue is closed with Vycril 2.0 wire, with simple stitches, and finally the skin is closed with nylon thread 3.0, with Donatti-type stitches. A simple dressing on the surgical wound is changed 24 hours before discharge. Antibiotic therapy is performed with intravenous 1 g cefazolin (EV) within 24 hours of the procedure. To use at home, dipyrone is prescribed 1 g orally (VO) every six hours for five days, and tramadol 50 mg VO in a rescue manner every eight hours.

## RESULTS

In the period studied, 17 patients were operated with the MIPO with locking technique. We excluded two patients due to loss of follow-up, thus remaining 15 patients as the object of analysis. No case required conversion of the closed to open technique.

Most patients were male (93%) and had fractures on the left side (80%). The mean age was 31.8 ± 12.7 years. According to the AO/OTA classification, six (40%) were type A, four (26.7%) type B, and five (33.3%) type C. Table 1 shows the general characteristics of the sample.

**Table 1:** General characteristics of the sample.

Characteristics	
**Age - years old (sd)**	31.8 (12.7)
**Sex - n (%)**	
Male	14 (93.3)
Female	1 (6.7)
**Affected side - n (%)**	
Right	3 (20.0)
Left	12 (80.0)
**AO classification - n (%)**	
A1	2 (13.3)
A2	4 (26.7)
B1	1 (6.7)
B2	2 (13.3)
B3	1 (6.7)
C1	3 (20.0)
C2	1 (6.7)
C3	1 (6.7)
**Fracture time - days (sd)**	8 (1.8)

sd: standard deviation.

Functional evaluation by the UCLA scale at 12 months showed a median of 35 (interquartile interval - IQR 2). The median of time of surgery was 54.8 minutes. Table 2 shows the data.

**Table 2:** Functional evaluation by the scale of the University of California in Los Angeles at 12 months and time of surgery.

	Mean	sd	Median	IQR
**UCLA scale**	34.1	1.2	35	2
**Time of surgery (minutes)**	54.8	21.6	50	35

UCLA: University of California in Los Angeles; sd: standard deviation; IQR: interquartile range.

All cases presented bone consolidation, which was found at three months in all 15 patients (100%).

In the evaluation of complications, three patients (20%) presented minor complications, one (6.7%) had anterior paraesthesia of the clavicle, and two (13.3%) evolved with prominence of the plate, but it was not necessary to remove the synthesis material. No patient presented hypertrophic scarring or skin irritation. Only one patient presented a major complication (6.7%) with surgical scar dehiscence, thus cleaning, debridement, and suture were performed in the operating room (Table 3). No other major complications were observed.

**Table 3:** Complications.

	n	%
Suture dehiscence*	1	6.7
Paresthesia**	1	6.7
Plate prominence**	2	13.3

*major complication; **minor complication.

## DISCUSSION

In this study, the minimally invasive treatment of displaced fractures from the clavicle had excellent clinical and radiographic results, with complete fracture healing in all cases. At the same time, the rate of complications was low compared to studies that used open plate fixation. ^(^ ^3^ The mean values found in the functional evaluation by the UCLA scale (34 points) are comparable to those described by Sohn, Kim, and Shon, ^(^ ^8^ who observed a mean of 33 in a retrospective study with 19 patients subjected to MIPO with locking. Treatment with open osteosynthesis also has excellent results. The Canadian Orthopedic Trauma Society reports 96 points according to the Constant-Murley scale and 5.2 by the DASH scale.^3^ Jiang and Qu, ^(^ ^7^ observed excellent results with both approaches, without statistically significant differences in the only randomized study comparing the conventional technique with the minimally invasive technique.

All patients in our series presented fracture healing at three months. The Canadian Orthopedic Trauma Society also describes 100% fracture healing in the group of 62 patients undergoing open osteosynthesis. ^(^ ^3^ Sohn, Kim, and Shon^8^ describe one case (3%) of pseudarthrosis in the 32 patients subjected to MIPO with locking, and no case in the group subjected to osteosynthesis openly, but without statistically significant difference.

In this study, the average surgery time was 54.8 minutes, shorter than that reported by Sohn, Kim, and Shon, ^(^ ^8^ with 77.2 minutes of average in the MIPO and 87.5 in open reduction and internal fixation (ORIF), but similar to that of Jiang and Qu, ^(^ ^7^ which report an average of 60 minutes in both techniques. Due to a learning curve at the beginning, to reproduce any minimally invasive technique is a greater difficulty; however, by acquiring a certain experience with a certain number of cases, surgery tends to occur faster than the conventional ORIF technique.

As minor complications, we had only one case (6.7%) that evolved with local paresthesia. Low rates of this complication are reported by other authors who evaluate MIPO. ^(^ ^7^ ^), (^ ^8^ Jiang and Qu. ^(^ ^7^ report two cases (6%) of paresthesia in the MIPO group, against 10 (31%) in the group subjected to MIPO with locking. On the other hand, Sohn, Kim, and Shon, ^(^ ^8^ report that none of the 19 patients subjected to MIPO with locking developed this complication. However, You, Wu, and Wang^11^ describe 11 cases (28.9%) in patients undergoing MIPO with locking. A possible explanation for the index of this complication is that we performed a small access (1.5 cm), focused on the distal orifices of the plate, thus preserving the branches of the supraclavicular nerve as much as possible. Comparative studies observe significantly lower values of this complication in patients operated by the MIPO technique, compared to patients operated by a crude technique. ^(^ ^7^ ^), (^ ^8^ You, Wu, and Wang^11^ report incidence of 29% vs 69%, whereas Jiang and Qu. ^(^ ^7^ 6% vs. 28%, demonstrating the greater safety of the minimally invasive technique related to this outcome. Two patients in our series (13.3%) presented prominence of the synthesis material, who did not need to be subjected to a new approach to implant removal, since the patients were satisfied with the result. The values obtained in this study agree with those reported by other authors. ^(^ ^9^ Sohn, Kim and Shon^8^ reported two cases (10%) among those subjected to the MIPO technique with this complication, a value lower than that observed in the group subjected to the conventional technique, that is, three cases (21%).

As major complications, we had one patient (6.7%) who evolved with suture dehiscence, and, thus, we needed a new approach in the operating room, where local cleaning was performed with a new closure of the subcutaneous tissue and skin, and osteosynthesis was not addressed. The Canadian Orthopedic Trauma Society describes this complication in three cases (5%) of patients subjected to ORIF with plate. ^(^ ^3^ Among the studies evaluating MIPO, we did not find reports of suture dehiscence.

We observed no type of synthesis failure, such as loosening or implant breakage. Thus we associated this failure with the fact that we use only blocked and unconventional plates (3.5 mm CPD and 3.5 mm reconstruction). Some biomechanical studies show the superiority of locking plates compared to conventional plates, although *in vivo* studies that present such differences are scarce. ^(^ ^12^ Mendes et al. ^(^ ^13^ ^)^ conducted a longitudinal observational study in comminuted fractures of the clavicle subjected to conventional MIPO, reporting two synthesis failures in 32 approaches, but no implant breaks or fatigues, such as Silva et al. ^(^ ^6^ in their comparative study on the surgical treatment of fractures displaced from the clavicle using conventional plates or intramedullary nail, without the break of the plate when this was the treatment option used.

Studies show that fractures with deviation and without contact between fragments present better results when treated surgically. ^(^ ^3^ ^), (^ ^14^ ORIF with plate is considered the gold standard of osteosynthesis today. ^(^ ^14^ Nevertheless, the results of studies evaluating MIPO allow us to say that this is a safe treatment option, with good clinical results and high consolidation rate. ^(^ ^7^ ^), (^ ^8^ ^), (^ ^11^

This study has some limitations. First, we cannot claim the superiority of this method over the gold standard due to the absence of a control group. Moreover, this study has a small casuistry and does not compare other techniques, open or minimally invasive. Finally, we used the UCLA scale as a clinical outcome, without specifically evaluating outcomes such as pain, range of motion, and quality of life.

This is one of the few studies on MIPO with locking in the treatment of fractures of the middle third of the clavicle, evaluating functional and radiographic results in a standardized manner. We consider this technique reproducible, safe, and viable, as an alternative to open osteosynthesis.

## CONCLUSION

MIPO with locking had excellent results according to the UCLA scale, fracture healing in all cases, and low rate of complications.
